# Changes in homocysteine and non-mercaptoalbumin levels after acute exercise: a crossover study

**DOI:** 10.1186/s13102-023-00656-w

**Published:** 2023-04-17

**Authors:** Akiho Shinagawa, Tomoki Yamazaki, Ayako Minematsu, Naho Serizawa, Yuri Hosoi, Yusuke Ninomiya, Yuichi Miyakoshi, Tomohiro Yano, Masako Ota

**Affiliations:** 1 Graduate School of Food and Nutritional Sciences, Toyo University, 1-1-1, Izumino, Itakura-Cho, Oragun, Gunma 374-0193 Japan; 2 Institute of Life Innovation Studies, Toyo University, Oragun, Gunma 374-0193 Japan; 3 Department of Ophthalmology, Keio University School of Medicine, Tokyo, 160-8582 Japan; 4 Department of Nutritional and Health Sciences, Faculty of Food and Nutritional Sciences, Toyo University, Oragunn, Gunma 374-0193 Japan

**Keywords:** Acute exercise, Homocysteine, Non-mercaptoalbumin ratio, Oxidative stress

## Abstract

**Background:**

Acute exercise is one factor that increases blood homocysteine levels, and elevated homocysteine levels cause oxidative stress. Albumin, which is abundant in blood, is an antioxidant, and the redox state of albumin is used as an index of oxidative stress in blood. This study aimed to assess the effect of acute exercise on plasma homocysteine levels and the blood non-mercaptoalbumin/mercaptoalbumin ratio as an oxidative stress marker.

**Methods:**

This study used a crossover design with exercise and control conditions. Under exercise conditions, a bicycle ergometer was used to perform 40 min of transient constant-load exercise at 65% heart rate reserve. Under control conditions, participants rested for 40 min. Blood was collected before, 30 min after, and 90 min after exercise, and at the same time points under control conditions. Samples were analyzed for the homocysteine concentration and non-mercaptoalbumin/mercaptoalbumin ratio.

**Results:**

The results revealed that a 65% heart rate reserve and 40 min of acute exercise increased plasma homocysteine concentration and non-mercaptoalbumin ratio. In the intra-condition comparison, the plasma Hcy concentration was significantly increased at Post 30 min (+ 0.83 ± 0.70 µmol/L, *P* = 0.003) compared with that at Pre in the exercise condition. Furthermore, 90 min after exercise, the blood non-mercaptoalbumin ratio was significantly increased (+ 0.35 ± 0.71%, *P* = 0.030) compared to Pre.

**Conclusion:**

These results indicate that the plasma Hcy concentration first increased, and then the non-mercaptoalbumin/mercaptoalbumin ratio increased as the elevated state was maintained. This study revealed that 65% heart rate reserve, 40 min of acute exercise increased plasma Hcy concentration and non-mercaptoalbumin ratio.

## Background

Homocysteine (Hcy) is an intermediate product of methionine metabolism with a thiol group [1]. The standard plasma Hcy concentration in humans is 3–15 μM [2]. Plasma Hcy concentration is increased by decreased activity of methylenetetrahydrofolate reductase (MTHFR) (due to genetic deficiency) or mutation [3], deficiency of cystathionine β synthase (CBS) [4]. The single nucleotide polymorphism C677T of the gene encoding MTHFR has been shown to reduce the activity of MTHFR. This MTHFR C677T decreases to 65% for CT type and 30% for TT type when the enzyme activity of CC type is 100%. This decrease in enzyme activity reduces the turnover of Hcy, leading to an increase in blood Hcy concentration. CBS also causes an increase in blood Hcy concentration by a similar mechanism [3–5]. Folic acid plays an important role in Hcy metabolism as it promotes the conversion of Hcy to methionine. Epidemiological studies have also shown that folic acid supplementation decrease plasma Hcy levels [6]. In addition, vitamin B2 is also involved in Hcy metabolism. Vitamin B2 is a precursor of flavin adenine dinucleotide, a coenzyme of MTHFR, and has been shown to be an independent determinant of plasma Hcy concentration [7]. Elevated plasma Hcy levels have arteriosclerosis-promoting and thrombosis-stimulating effects, and hyperhomocysteinemia is an independent risk factor for cardiovascular and neurodegenerative diseases [8, 9].

Plasma Hcy levels have been reported to decrease with long-term, regular exercise [10], while there are numerous reports that Hcy levels increase after acute exercise. In these reports, acute exercise included bicycling [11, 12], walking and running on a treadmill [13–15], running in a marathon or half marathon [16–18], participating in a triathlon [19], performing resistance exercise [20], and other activities. However, few studies have explored the relationship between plasma Hcy concentration and exercise conditions in which participants are subjected to the same load, even within the same sport [21]. The participants must exercise with the same load to accurately assess the change in plasma Hcy concentration after acute exercise.

The detailed mechanism by which plasma Hcy concentrations increase after acute exercise is unclear. However, several mechanisms have been considered, including an energy-independent metabolism derived from exercise. It is thought that the increase in metabolic demand due to exercise promotes the metabolism of methylated substrates (e.g., creatine, acetylcholine, RNA, and DNA), and that the plasma Hcy concentration increases to compensate for this [22]. In particular, creatine synthesis consumes more methyl groups [23] and may result in a sustained increase in plasma Hcy levels.

Albumin, the most abundant protein in plasma, accounts for approximately 50% or more of the total protein in plasma, and the standard plasma concentration is 0.6–0.75 mM [24]. The thiol group at the Cys34 residue of albumin is involved in the transport of various substances in the body, and this thiol group is susceptible to oxidative modification. Therefore, the ratio of non-mercaptoalbumin (the oxidized form of albumin) to mercaptoalbumin in the blood is used as a systemic (whole blood) oxidative stress biomarker [25]. Furthermore, non-mercaptoalbumin is reportedly associated with and elevated after exercise [26, 27].

After transient exercise, the plasma Hcy concentration increases due to the increased required number of methylated molecules, including acetylcholine and creatine. Moreover, the blood non-mercaptoalbumin/mercaptoalbumin ratio increases with the oxidation of the Cys34 residue of albumin. However, no study has explored these changes over time. Therefore, this study aimed to understand the dynamics of the blood non-mercaptoalbumin/mercaptoalbumin ratio when the plasma Hcy concentration increases during acute exercise using a relatively similar load amongst participants.

## Methods

This study used a crossover design and included exercise and control (non-exercise) conditions; the two trials were separated by at least one week.

The participants’ exercise conditions were 65% heart rate reserve (HRR) using a bicycle ergometer (Ergomedic 828E, Monark Exercise AB, Vansbro, Sweden) and a constant-load exercise for 40 min, with the number of revolutions maintained at 60 revolutions per minute. This exercise condition was set with reference to a previous study [21]. A gradual load bicycle exercise test was conducted in advance, and a 65% HRR load was individually set based on the set exercise load (measured in kiloponde [kp]) and the measured heart rate (HR). In the gradual load bicycle exercise test, after resting for 2 min, the exercise load was increased by 0.5–1.0 kp every 3 min, and a total of 12 min of bicycle exercise was performed. The number of revolutions was maintained at 60 revolutions per minute. HR was recorded during the last 15 s of each exercise load. While at rest for the first 2 min, the HR was recorded for 15 s each during the 45–60 s and 105–120 s timepoints. From the recorded results, the exercise load (kp) was calculated for each participant using the Carbonen method (exercise intensity (% HRR) = [(HR during exercise-HR at rest)/(maximum HR-HR at rest)] × 100). Under control conditions, which were performed one week after the exercise conditions, the same participants rested the same amount of time as they exercised. During rest, sleep and food were excluded and participants were monitored by the examiner.

### Participants

This study was conducted in 2018 following the guidelines of the Declaration of Helsinki and was approved by the Committee for the Ethical Guidelines for Medical and Health Research Involving Humans Subjects at TOYO University (approval number: TU2018-015, TU2019-024). Written informed consent was obtained from all participants after they were informed of all the risks, discomforts, and benefits of the study. Participants were recruited from Toyo University, Japan. The study enrolled 44 healthy individuals aged > 20 (29 men and 15 women) who had not received any medication. The exclusion criteria were as follows: (1) inability to complete the transient constant-load exercise equivalent to 65% HRR (n = 10), (2) suspected hyperhomocysteinemia (n = 1), (3) inability to provide all six blood samples over time (n = 3), and (4) did not complete the analysis of blood components due to hemolysis (n = 12), (5) analysis of blood non-mercaptoalbumin/mercaptoalbumin ratio was not completed (n = 4). The final sample included 14 participants (10 men and 4 women). The sample size was calculated at a power of 80% using α = 0.05. The effect size of Hcy was 0.6 µmol/L and was necessary of a sample of 11 to validate results. The number of participants in this study met the calculated value.

### Blood sampling and analyses

By venipuncture, the amount of blood collected at one time was 10 ml. The first blood collection for each condition was performed between 8 and 9 am, before exercise (Pre), and after a 12-h fast. Further blood was collected at two points, 30 min after exercise (Post 30 min) and 90 min after exercise (Post 90 min). Blood was collected at the same time points under the control conditions. Whole blood was placed in an EDTA-2 K tube to measure of plasma Hcy concentration, plasma vitamin B2 concentration, and plasma folic acid concentration and centrifuged (3000 rpm, 10 min) within 20 min. Whole blood for measuring the blood non-mercaptoalbumin/mercaptoalbumin ratio was collected in a citrate tube and centrifuged (3000 rpm, 10 min) within 20 min. Blood samples were stored at -30 °C until analysis. The plasma Hcy concentration was analyzed using high performance liquid chromatography. The analysis conditions are presented in Table 1. The blood non-mercaptoalbumin/mercaptoalbumin ratio was outsourced to LSI Medience Corporation, Japan, for analysis. The plasma vitamin B2 concentration was quantified by microbiological analysis using ID-Vit ^®^ vitamin B2 in serum (Immundiagnostik AG, Bensheim, Germany). The plasma folic acid concentration was analyzed using the Folic Acid Enzyme-linked Immunosorbent Assay Kit (CELL BIOLABS, INC., USA).

**Table 1:** Analytical conditions for plasma homocysteine level by high-performance liquid chromatography

Pump	EP-700 (LIQUID CHROMATOGRAPH PUMP/Eicom)
Auto Sampler	M-514 (Eicom)
Detector	Eicom ECD-700 (Eicom)
Working Electrode	Eicom WE-AU (Eicom)
Precolumn	COSMOSIL Guard Cartridge 5PFP 4.6ID × 10 mm (COSMOSIL)
Column	Eicom-3OSD 3.0φ × 150 nm (Eicom)
Column Temperature	25 °C
Buffer	99% 0.1 M Sodium phosphate buffer (pH 2.5), 1% Methanol, 170 mg/L Sodium octansulfonate, 5 mg/L EDTA-2Na
Flow rate	500 µL/min

Body composition was measured using a bioimpedance device (InBody770, InBody Japan Co., Tokyo, Japan) to determine body weight, body fat mass, and muscle mass at the time of each condition. Height was measured using a manual height meter (BSM170B, InBody Japan Co., Tokyo, Japan).

### Statistical analysis

All statistical analyses were carried out with SPSS statistical software version 26 (IBM Japan, Tokyo, Japan). The average value and standard deviation were calculated for all measures.

Elapsed time (within conditions) and the presence or absence of acute exercise (between conditions) were factors for investigating the temporal effects of exercise on the plasma Hcy concentration, blood non-mercaptoalbumin/mercaptoalbumin ratio, plasma vitamin B2 concentration, and plasma folic acid concentration. A two-way analysis of variance (ANOVA) was performed using repeated measurements. If a significant difference was found in the inter-condition comparison, a paired t-test or Wilcoxon signed-rank test was performed to identify the cause of the significant difference. If a significant difference was found in the intra-conditional comparison, a one-way ANOVA or Friedman test by repeated measurement was performed. All tests were two-sided, and the significance level was set at *P* = 0.05.

## Results

**Fig. 1:**
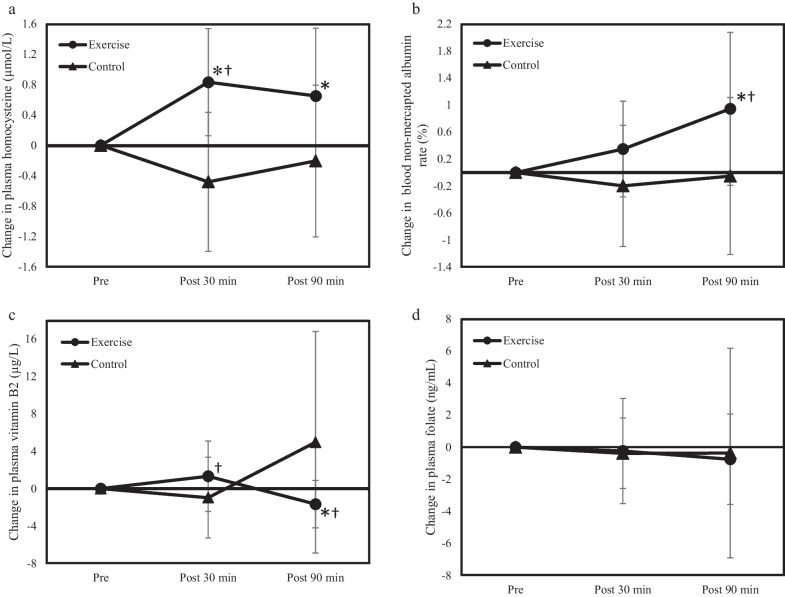
Changes in the level of each component in plasma and blood. The level of Pre was used as the baseline, and the amount of change at Post 30 min and Post 90 min are shown. Pre indicates before exercise, after 12 h of fasting. Post 30 min and Post 90 min indicate after 30 min and 90 min of exercise, respectively. Changes in (**a**) homocysteine, (**b**) non-mercaptoalbumin/mercaptoalbumin ratio, (**c**) vitamin B2, and (**d**) folate are shown. ^*^P < 0.01, significantly different from control at the same time point. ^†^*P* < 0.01, significantly different from Pre

The final participants were 14 (10 men and 4 women). The participants were aged 21.6 ± 0.7 years, weighed 61.5 ± 10.9 kg, were 169.2 ± 8.4 cm in height, had a body mass index of 21.4 ± 3.1 kg/m^2^, a muscle mass of 46.4 ± 8.9 kg, and body fat mass of 12.3 ± 4.6 kg. There was no change in the participants’ physical characteristics between the exercise and control conditions. A two-way ANOVA was performed by repeated measurements to determine the exercise effect (between conditions) and time effect (within conditions) on the plasma Hcy concentration, blood non-mercaptoalbumin/mercaptoalbumin ratio, and plasma folic acid and plasma vitamin B2 levels. Figure 1a–d shows the amount of change in each measurement at Post 30 min and Post 90 min compared to the value at Pre of 0.

An interaction between the exercise effect and the time effect was observed (*P* = 0.000) in the amount of change in plasma Hcy concentrations (Fig. 1a). Inter-condition comparisons showed a significant difference between the exercise and the control conditions at Post 30 min and Post 90 min (*P* = 0.001 and *P* = 0.018, respectively). In the intra-condition comparison, there was a significant increase at Post 30 min (*P* = 0.003) compared with that at Pre in the exercise condition; however, there was no change in the control condition.

There was also a significant difference in the interaction between the exercise and time effects on the amount of change in the blood non-mercaptoalbumin/mercaptoalbumin ratio (Fig. 1b, *P* = 0.026). Inter-condition comparisons showed a significant difference between the exercise and control conditions at Post 30 min (*P* = 0.035). Under exercise conditions, there was a significant increase at Post 90 min compared to that at Pre (*P* = 0.030); however, there was no significant difference under control conditions.

An interaction between the exercise and time effects was observed (*P* = 0.014) in the amount of change in plasma vitamin B2 concentrations (Fig. 1c). Inter-condition comparisons showed a significant difference between the exercise and control conditions at Post 90 min (*P* = 0.022). In the intra-conditional comparison, under exercise conditions, a significant increase was observed at Post 30 min (*P* = 0.002) and a significant decrease was observed at Post 90 min (*P* = 0.001) compared with that at Pre.

There was no significant difference between the conditions regarding the amount of change in plasma folic acid concentrations (Fig. 1d).

There were no significant differences in the four blood components between men and women in the amount of change in blood components at the blood sampling points after exercise.

## Discussion

This study aimed to investigate the effect of acute exercise on plasma Hcy concentration and blood non-mercaptoalbumin/mercaptoalbumin ratio. The data show that plasma Hcy concentration increase 30 min after acute exercise of 65% HRR and blood mercaptoalbumin/mercaptoalbumin ratio increases after 90 min. From this result, it was clarified that in the acute exercise of 65% HRR, the plasma Hcy concentration first increased, and when the increased state was maintained, the blood non-mercaptoalbumin/mercaptoalbumin ratio increased.

Regarding the association between acute exercise and Hcy, König et al. reported that intense acute exercise increased plasma Hcy concentration in healthy young men aged 24–39 years [28]. There is also a report that acute aerobic exercise at an intensity of 70% of maximal HR increased plasma Hcy concentration [13]. For Hcy and training, the results of aerobic and resistance training are different. A meta-analysis showed that resistance training lowered plasma Hcy levels, while aerobic training had no effect [29]. Therefore, results vary depending on the type and intensity of the exercise and the length of the program. The exact mechanism that gives rise to these differences remains unknown. In this study, we found that plasma Hcy concentration increased even with 65% HRR intensity exercise, as in the previous study with reference to the exercise model.

To date, the levels of non-mercaptoalbumin have been reported to increase after competing in various sports; however, the exercise intensity exerted by participants has not been specified [27, 30]. Even when the same type of exercise is performed, the tightness of the exercise is not the same depending on the subject’s physical ability. In order to remove this potential difference in physical performance, we believe it is necessary to expose the participants to the same relative load. For these reasons, we conducted the experiment using an exercise model of 65% HRR and 40 min, which differs from previous studies. We believe that using this exercise model allowed us to clarify the intensity of the exercise applied to the participants and to obtain more accurate results. In this study, participants exercised with the same relative load, and it is a novel finding that the blood non-mercaptoalbumin/mercaptoalbumin ratio increases with exercise equivalent to 65% HRR. From this result, it is inferred that the previous studies on an intensive training camp for kendo [27] and the 103 km mountain ultramarathon [30], which reported an increase in the blood non-mercaptoalbumin/mercaptoalbumin ratio, had a load equal to or higher than that in this study.

Furthermore, the mechanism of the increase in the blood non-mercaptoalbumin/mercaptoalbumin ratio after exercise has not been clarified. Hcy may be a factor in elucidating this mechanism. When Hcy cannot be metabolized intracellularly, it moves into the blood. Most Hcy is bound to proteins in the blood [31]. Reactive oxygen is generated when Hcy and protein bind. These reactive oxygen species are normally removed by scavenging enzymes, such as superoxide dismutase and low molecular weight antioxidants, to protect the body from injury. However, when equilibrium (homeostasis) is disturbed, these reactive oxygen species also damage surrounding cells due to their high reactivity, causing dysfunction [14]. One of these dysfunctions is the oxidative modification of the thiol group of the Cys34 residue of albumin, which increases the non-mercaptoalbumin/mercaptoalbumin ratio. In other words, it is considered that the acute exercise of 65% HRR increased the amount of active oxygen generated and the homeostasis temporarily collapsed as the plasma Hcy concentration increased. The rationale for this is that the blood non-mercaptoalbumin/mercaptoalbumin ratio increased by 0.35% 30 min after exercise and 0.94% after 90 min of exercise compared to before the start of exercise. After exercise, the relative amount of reduced mercaptoalbumin ratio decreased, and non-mercaptoalbumin/mercaptoalbumin ratios increased compared with that before exercise. The abundance ratio can be expressed using the following equation:

$${\text{Non}} - {\text{mercaptoalbumin ratio}} = \frac{{{\text{HSA}} - 34{\text{Cys}} - {\text{S}} - {\text{S}} - {\text{Cys}}}}{{{\text{HSA}} - 34{\text{Cys}} - {\text{S}} - {\text{S}} - {\text{Cys }} + {\text{ HSA}} - 34{\text{Cys}} - {\text{SH}}}}$$

The result supports that the binding of Hcy to albumin increases over time [32].

One of the causes of the increase in oxidative stress by acute exercise is the increase in blood flow and oxygen intake of tissues and active muscles. On the other hand, continuous training is thought to increase the antioxidant enzyme activity of the trained muscles and relieve oxidative stress during exercise [33]. Low levels of transient oxidative stress increase provide benefits, while high levels increase muscle damage and benefit diminish [33]. Oxidative stress is also associated with various diseases, including lifestyle-related conditions, but the effects of exercise-induced oxidative stress on these remain controversial. In this study, the blood non-mercaptoalbumin/mercaptoalbumin ratio, used as an oxidative stress marker increased significantly after 90 min of exercise. To quickly recover from muscle fatigue, it is desirable to quickly return the blood non-mercaptoalbumin/mercaptoalbumin ratio to that at rest. Therefore, it is preferred that the time during which the plasma Hcy concentration rises from the resting concentration is as short as possible.

Based on this result, it is hypothesized that an increase in Hcy is involved in one of the factors that increase the blood non-mercaptoalbumin/mercaptoalbumin ratio after acute aerobic exercise. It may be possible to mitigate increased production as plasma Hcy levels can be reduced by nutrient supplementation. Supplementation with creatine reduces the increase in plasma Hcy concentration after high-intensity exercise in animal models [34]. In this study, it was suggested that the increase in plasma Hcy concentration may be involved in the increase in post-exercise oxidative stress marker (non-mercaptoalbumin/mercaptoalbumin ratio), which may help to reduce the increased oxidative stress production after exercise. We believe this is one finding that could benefit the health of those who exercise.

It was also clarified that vitamin B2 increased significantly 30 min after and decreased 90 min after exercise when the pre-exercise was time point 0. A previous study confirmed that blood vitamin B2 levels increased significantly immediately after exercise when measured before and after walking 100 km [35]. In contrast, there was a report showing that vitamin B2 status was not affected after acute exercise using a bicycle erfometer [36]. Exercise requires more energy turnover and stresses the metabolic pathways that produce energy. Exercise may increase the need for vitamin B2, which is essential for energy production [37]. The decrease in blood vitamin B2 levels 90 min after exercise in this study may be due to its increased need due to its metabolism. Furthermore, since vitamin B2 has a role as a coenzyme of MTHFR, it is possible that it was used to efficiently metabolize increased Hcy during exercise.

Blood folic acid levels were not affected by exercise. Plasma folic acid levels did not change after a young soccer player performed an acute sprint exercise [14]. The present study agreed with this result.

This study had two limitations. First, the direct relationship between the blood non-mercaptoalbumin/mercaptoalbumin ratio and blood albumin concentration could not be observed. It has been suggested that the blood non-mercaptoalbumin/mercaptoalbumin ratio may be an indicator of oxidative stress after acute aerobic exercise. However, to clarify the mechanism, blood albumin should be quantified in the future. Second, there are multiple factors that increase the blood non-mercaptoalbumin/mercaptoalbumin ratio, not just the increase in plasma Hcy concentration, and these factors have not been studied. In future studies, we hope that after quantifying the albumin in the blood, whether a direct relationship exists between the ratio and concentration of non-mercaptoalbumin in blood will be clarified.

## Conclusion

These results indicate that after 65% HRR and 40 min of transient constant-load exercise, the plasma Hcy concentration first increased, and then the non-mercaptoalbumin/mercaptoalbumin ratio increased as the elevated state was maintained. We could establish a novel acute exercise model that increased plasma Hcy concentration and the blood non-mercaptoalbumin/mercaptoalbumin ratio. Since blood non-mercaptoalbumin is a systemic oxidative stress marker, it was found that acute exercise equivalent to 65% HRR for 40 min increased systemic oxidative stress. Transient exercise equivalent to 65% HRR is moderate intensity exercise, not high-intensity exercise. Specifically, it is inferred that the oxidative stress of the whole body increases without high-intensity exercise like that of an athlete, which may be clinically significant. In the future, larger-scale research is needed to confirm our findings and determine whether suppressing the increase in plasma Hcy after exercise will suppress the increase in the blood non-mercaptoalbumin/mercaptoalbumin ratio.

## Data Availability

The datasets used and/or analyzed during the current study are available from the corresponding author on reasonable request.

## Abbreviations

ANOVA: Analysis of variance
BMI: Body mass index
CBS: Cystathionine β synthase
Hcy: Homocysteine
HPLC: High-performance liquid chromatography
HR: Heart rate
HRR: Heart rate reserve
kp: Kilopond
MTHFR: Methylenetetrahydrofolate reductase
Pre: After 12 h fasting and before exercise
Post 30 min: 30 Minutes after exercise
Post 90 min: 90 Minutes after exercise
